# Factors Influencing Collaborative Activities between Non-Professional Disaster Volunteers and Victims of Earthquake Disasters

**DOI:** 10.1371/journal.pone.0047203

**Published:** 2012-10-16

**Authors:** Tomoko Haraoka, Toshiyuki Ojima, Chiyoe Murata, Shinya Hayasaka

**Affiliations:** 1 Department of Regional Medical Management, Hamamatsu University School of Medicine, Higashiku, Hamamatsu, Shizuoka, Japan; 2 Department of Community Health and Preventive Medicine, Hamamatsu University School of Medicine, Higashiku, Hamamatsu, Shizuoka, Japan; 3 Section of Social Participation and Support, Department of Social Science, Center for Gerontology and Social Science (CGSS), National Center for Geriatrics and Gerontology (NCGG), Aichi, Japan; 4 Department of Sports and Health Science, Daito Bunka University, Higashimatsuyama, Saitama, Japan; Tehran University of Medical Sciences, Iran (Islamic Republic of)

## Abstract

**Background:**

Assistance from non-professional disaster volunteers (hereinafter, volunteers) is essential for disaster victims to recover physically and rebuild their lives; however, disaster victims in some areas are reluctant to accept assistance from volunteers. This study explored factors that may influence collaborative activities between volunteers and victims of earthquake disasters.

**Methods:**

From July to September 2008, a self-reporting questionnaire survey was conducted with all 302 leaders of neighborhood associations in a city within Niigata Prefecture at the time of the Niigataken Chuetsu-oki Earthquake in 2007. Each factor was determined based on the Health Belief Model. Multiple regression analysis was conducted, using collaborative activities as the objective variable.

**Results:**

From 261 valid responses received (response rate 86.4%), 41.3% of leaders collaborated with volunteers, and 60.2% of associations had residents who collaborated with volunteers. Collaboration with volunteers was significantly and positively related to perceived severity of an earthquake disaster (standardized partial regression coefficient β = 0.224, p<0.001) and neighborhood association activities during the earthquake disaster (β = 0.539, p<0.001). A positive and marginally significant relation was found between such collaboration and sense of coherence within a community (β = 0.137, p = 0.06), social capital (β = 0.119, p = 0.08), and perceived benefits (β = 0.116, p = 0.09).

**Conclusion:**

Collaboration between disaster victims and volunteers during the response to an earthquake may require the preemptive estimation of damage by residents during normal times and the enhancement of neighborhood association activities during a disaster. For residents to have such estimation abilities, public institutions should provide information related to anticipated disaster damage and appropriate disaster prevention training and education. In addition, residents should create a disaster prevention map with other residents. Lastly, promoting neighborhood association activities may require the participation of many residents in disaster drills and education as well as a preemptive discussion of neighborhood activities during a disaster.

## Introduction

The natural conditions of Japan, including its geographical position, geographical formations, and geology, mean it is prone to earthquake disasters. Japan is also left vulnerable to such disasters due to its aging population, weakening of community ties, overcrowding of cities, and depopulation of rural areas. In circumstances such as these, neighborhood associations, recognized as “groups formed by neighborhood ties” [1], conduct disaster prevention activities on a regular basis.

During previous earthquake disasters, neighborhood associations within towns in the disaster areas have played important roles in saving lives, evacuating residents, and providing living assistance immediately after the earthquake. Since the functioning capacity of these neighborhood associations is far from sufficient, they respond to disasters with the help of government, businesses, schools, and disaster volunteers. Unlike the government's response to standard and common needs which is characterized by limited personnel and points of assistance, disaster volunteers are capable of flexibly responding, in large numbers, to the diverse needs of victims. Their activities are an absolute necessity [2].

Disaster volunteers are classified from various perspectives. They can be classified according to their expertise, as either specialist disaster volunteers who possess professional skills, such as those found in the medical profession, or non-professional disaster volunteers who are average citizens with no particular professional skills and who implement support activities in the disaster areas [3]. The great majority of non-professional disaster volunteers (hereinafter, volunteers) in Japan have not participated in organized disaster prevention drills or received education during non-emergency times. Furthermore, a large number of them have no previous experience of volunteering in disaster areas.

Following the significant attention that the volunteer efforts after the Great Hanshin-Awaji Earthquake of 1995 attracted, it has now become common for large numbers of volunteers to rush to disaster sites and conduct relief activities immediately following disasters [4]. For the Great East Japan Earthquake that struck in March 2011, over 895,000 volunteers conducted activities in the three prefectures with disaster sites (Iwate, Miyagi, and Fukushima Prefectures) as of December 25, 2011 [5]. When a disaster occurs, volunteers go to the government-established disaster volunteer center at the disaster site and work from there without compensation. More specifically, disaster volunteer centers direct volunteers to conduct activities alongside those who have requested aid in accordance with the known needs of victims and neighborhood associations in the disaster area. In some cases, however, disaster victims have been reluctant to accept assistance from volunteers. For example, victims did not become accustomed to the presence of volunteers who came to provide support activities after the Northern Miyagi Earthquakes in September 2003 [6]. Likewise, in the Niigata Prefecture Chuetsu Earthquake in July 2004, many victims declined aid from volunteers, and there were many instances where victims did not seek assistance for themselves [7]. Subsequently, the Cabinet Office of the Government of Japan has been promoting the concept of “aid acceptance”, namely, the environment and the knowledge needed for victims to actively accept volunteers for recovery and reconstruction activities in disaster areas [8]. In addition, a previous study showed that cross sector collaboration (a partnership involving business, nonprofit organizations, philanthropies, and communities and/or the public as a whole), is necessary to compensate for a weakness in one sector and the administrative void in disaster correspondence [9]. For disaster victims to recover physically and promptly rebuild their lives, it is necessary for volunteers to be accepted by victims and “that victims and volunteers work together” (hereinafter, collaborative activities) based on equal relationships of mutual understanding and respect. However, factors that lead to such collaborative activities remain unclear. This study, therefore, aimed to reveal the contributing factors for carrying out collaborative activities between victims and volunteers during earthquake disasters.

## Materials and Methods

### Research materials

This study concerned the 6.6 magnitude Niigataken Chuetsu-oki Earthquake that occurred on July 16, 2007. All 302 leaders of neighborhood associations across an entire city in the disaster area in Niigata Prefecture at the time of the disaster were surveyed in July to September, 2008. A self-administered questionnaire was distributed to the leaders by mail. The leaders completed the questionnaire anonymously and returned it by mail.

We regarded the leaders in each community as representatives of victims on the basis of the results of a survey conducted by the city office in the previous year. Accordingly, the neighborhood association leaders were chosen by election, rotation, or via a lottery among all residents. Furthermore, 92.9% of neighborhood association leaders routinely talked with residents about local problems such as the environment, disaster prevention, and crime prevention during normal times [10]. The leaders were selected as study subjects because they themselves were disaster victims and were therefore well acquainted with their neighborhood and able to empathize with disaster victims.

This study used the Health Belief Model (HBM), a behavior modification theory, to systematically analyze the factors that contributed to carrying out collaborative activities between victims and volunteers during the earthquake disaster. HBM is a model which assumes that a person's perception and belief will affect his or her health behavior [11]. We also considered using other individual-level behavior change theories such as the Stage of Change Model, Precaution Adoption Process Mode, and Theory of Planned Behavior explain behavior modification through process and behavior intention [12] during study planning. The reasons for using HBM in the study are as follows. First, the HBM is a good fit for addressing problem behavior that evokes desirable concerns and also for clarifying components of the model (factors) to reinforce recommended behavior [12], [13]. Second, although it might be feasible to conduct a questionnaire survey on factors of HBM following a disaster, it was difficult to investigate by means of other models the detailed behavior process and the intention to perform recovery work when the earthquake struck.

The conceptual framework of collaboration activities when using HBM in this study was as follows (Figure S1). The perceived susceptibility and severity of earthquake disasters may enhance the recognition of the threat. Furthermore, gaps between perceived benefits of collaborative activities and perceived barriers may determine the collaborative activities. Recognition of the threat may then be affected by neighborhood activities, SC, and SOC, as well as by knowledge and experience. Finally, cues for action may promote collaborative activities.

Before conducting the present study, we visited the disaster site and conducted a small face to face unstructured pilot survey. Then, on the basis of the results, we examined and created the current survey items to reflect factors of the conceptual framework, which was based on HBM factors such as health actions, perceived susceptibility, perceived severity, perceived benefits, perceived barriers, cues for action, sociopsychological variables, and structural variables. Collaborative activity with volunteers was designated as health action. Sociopsychological variables were the four factors of neighborhood association activities, social capital (hereinafter, SC), sense of coherence (hereinafter, SOC) in the community, and individual SOC. Knowledge and experience were selected as structural variables. From the following survey items, 11 factors were generated. 1) Collaborative activity items examined initiatives for cooperation between neighborhood association leaders, residents, and volunteers, triggers for cooperative activity, as well as support for volunteer efforts by leaders and residents. 2) Perceived susceptibility items related to estimates for earthquakes. 3) Perceived severity items related to earthquake scenarios. 4) Perceived benefit items related to usefulness and outcomes. 5) Perceived barrier items related to feelings of resistance. 6) Cue to action items related to information. 7) Neighborhood association activity items related to the activities and circumstances of the town after the earthquake. Items on 8) SC and 9) SOC in the community were from the scale used in the extensive surveys carried out in the Aichi Gerontological Evaluation Study [14]. 10) Individual SOC items were taken from a shortened version of Antonovsky's SOC questionnaire, the SOC-UTHS scale (a three-item Tokyo University Health and Social Sciences version of the SOC scale) [15]. 11) Knowledge and experience items related to knowledge, experience, and participation in training on disaster prevention. Regarding factor reliability, Cronbach's alpha revealed a high value of 0.84 and a low level of 0.47 (Table S1). Furthermore, to establish the criterion-related validity of each factor, associated items were set as external criteria, and a Kendall test was run. Results showed a significantly association between factors and external criteria. (Table S2).

The study was conducted with approval from the Ethics Review Board of Hamamatsu University School of Medicine (No. 20-5).

### Analysis

First, for each of the 11 factors, principle component analysis was conducted to aggregate 2–9 items into a single-factor score. Principal component analysis was used because it is generally appropriate for computing a total score from multiple variables. The analysis determines the weighting coefficient of each variable (i.e., factor loading) so that the variance of composite variables is maximized [16], [17]. In the present study, we obtained the factor loading of 2–9 items of the 1st principal component for each factor. Then, a linear combination (i.e., sum of the product of the factor loading and the collected data of each item) was calculated, and the score of each factor was obtained.

Second, multiple regression analysis was conducted with collaborative activities as the objective variable and all other factors as explanatory variables. An adjusted standardized partial regression coefficient was calculated including explanatory variables of damage conditions and number of households as covariates. Associations were then examined. SPSS for Windows ver. 15.0 (SPSS Inc., Tokyo, Japan) was used for all data analysis.

## Results

There were 261 valid responses, for a valid response rate of 86.4%. Collaborative activities and each survey item are shown in Table 1. Males accounted for 98.9% of the neighborhood association leaders and the mean age of respondents was 68.6 (SD = 5.7). The mean number of households within an association was 113.5 (SD = 147.2).

**Table 1:** Collaborative activities and explanation on each survey item.

				n = 261
Factor	Items	n	(%)	Mean ± SD
Demographic characteristics*2	Sex of the neighborhood association leader (male)	258	(98.9)	
	Age of leader			68.6±5.7
	Number of households within a neighborhood			113.5±147.2
	Damage was severe*3	114	(43.7)	
	Leader collaborated with non-professional volunteers	109	(41.8)	1.4±1.9*4
Collaborative activities*2	Leader assisted non-professional volunteers' activities	126	(48.3)	1.5±1.5*4
	Community residents collaborated with non-professional volunteers	157	(60.2)	3.1±3.1**4
	Community residents assisted non-professionalvolunteers	145	(55.6)	1.6±1.4*4
	Reasons to initiate collaborative activities between residents and non-professional volunteers			0.9±0.9***4
Perceived susceptibility*1 *5	Anticipated small-size earthquake	121	(46.4)	
	Anticipated a medium to large sized earthquake	40	(15.3)	
Perceived severity*1 *5	Anticipated human suffering due to earthquake	55	(21.1)	
	Anticipated earthquake damage to buildings	79	(30.3)	
	Anticipated that the earthquake would affect my life	99	(37.9)	
Perceived benefits*1 *5	Expected activities would sort out things earlier	135	(51.7)	
	Expected activities would help restoration	178	(68.2)	
	Expected would be able to respond to problems earlier through activities	101	(38.7)	
Perceived barriers*1 *5	Resistant to engaging in collaborative activities	76	(29.1)	
	Questioning the extent of collaborative activities	102	(39.1)	
	Reticent about revealing personal information	103	(39.5)	
Cues to action*2 *5	Knew about non-professional volunteers from media	237	(90.8)	
	Knew about non-professional volunteers from government information	192	(73.6)	
	Knew about non-professional volunteers from personal communications	214	(82.0)	
	We performed activities to respond to disaster within a neighborhood	251	(96.8)	7.2±3.0**4
Neighborhood associations activities*2	Work as a leader was difficult	206	(78.9)	
	There was an evacuation area in my neighborhood	124	(47.5)	
	I was involved with management of the evacuation area	138	(52.9)	
	Non-professional volunteers came to my neigborhood	153	(58.6)	
	Less than 40% of neighborhood association members have resided in the neighborhood for 20 years	18	(6.9)	
	There was a voluntary disaster-prevention organization within our neighborhood	83	(31.8)	
Social capital*1	I think people can be trusted	106	(40.6)	
	I think people take advantage of others	21	(8.0)	
	I think people try to be helpful to others	113	(43.3)	
Community's sense of coherence*1 *7	Large event, overcoming it			2.8±1.3
	Calm decision making in response to a large event			3.6±1.0
	Something acquired through a large event			3.8±0.9
	Community problems, solution by the community			2.7±1.1
	Community problems, residents' thoughts on solution			3.6±0.9
	Communal problems, strengthening trusting relationships			3.8±0.9
Individuals' sense of coherence*1 *6	Finding solutions for problems			4.9±1.4
	Values working on problems			5.3±1.3
	Understanding and predicting problems			4.7±1.3
	Past experience of earthquakes	140	(53.6)	
	Knowledgeable about roles as a leader after the earthquake disaster	202	(77.4)	
Knowledge and experiences*1	Participated in lectures and workshops	136	(52.1)	
	Participated in disaster prevention training	121	(46.4)	
	Knew areas and process for evacuation	237	(90.8)	
	Knowledgable about what to be prepared for in normal times	215	(82.4)	
	Proactive about non-professional volunteer activities	98	(37.5)	
	Had concerns about non-professional volunteer activities	41	(15.7)	
	Experience of volunteering	97	(37.2)	

*1 Thoughts and actions prior to the earthquake disaster;

*2 Actions and circumstances during the earthquake disaster;

*3 1. Total n (%) of responses of “1. Very severe” and “2. Relatively severe”;

*4 Mean±SD of number of responses on choices on 7 items;

**4 Mean±SD of number of responses on choices on 15 items;

***4 Mean±SD of number of responses on choices on 4 items;

*5 Total n (%) of responses “1. Had thought (felt, known) about it” and “had thought about it somewhat”;

*6 Mean±SD of number of resoponses on choices on a 7-point rating responses;

*7 Mean±SD of number of responses on choices on a 5-point rating scale.

As to perceived susceptibility to an earthquake before the disaster, 46.4% of respondents had anticipated a small earthquake. As to perceived severity, 37.6% predicted that an earthquake would affect their lives. With regard to perceived benefits, 68.2% had thought that collaborative activities with volunteers would be helpful in recovery efforts following a disaster. In terms of perceived barriers, 39.5% indicated reluctance in revealing personal information to volunteers, and as to cues to action, 90.8% had obtained information about volunteers from the media.

During the earthquake disaster, 96.8% of neighborhood associations responded to address earthquake damage, and volunteers arrived in 58.6% of neighborhoods. Collaborative activities with volunteers were conducted by 41.8% of all neighborhood association leaders and 60.2% of neighborhoods had residents who performed such activities.

Factor loadings for each factor item score are shown in Table 2. Principal component analysis was conducted for each item and calculations of the factor loadings of the first principal components yielded positive values. The factor loading values of the nine factors, excluding “Neighborhood Association Activities” and “Knowledge and Experience”, were mostly the same at around 0.8.

**Table 2:** Factor loading for each factor score.

Factors	Items	Factor loadings
Scores for collaborative activities	Collaborative activities between leaders and non-professional volunteers	0.80
	Leaders' assistance for non-professional volunteers' activities	0.89
	Collaborative activities between residents and non-professional volunteers	0.81
	Residents' assistance for non-professional volunteers' activities	0.88
	Reasons to initiate collaborative activities between residents and non-professional volunteers	0.84
Scores for perceived susceptibility	Anticipating small-size earthquake	0.88
	Anticipating a medium to large sized earthquake	0.88
Scores for perceived severity	Anticipating human suffering due to earthquake	0.78
	Anticipating earthquake damage to buildings	0.88
	Anticipating that the earthquake would affect my life	0.89
Scores for perceived benefits	Effectiveness for sorting out things	0.81
	Helpfulness for restoration	0.87
	Early responses to troubles	0.82
Scores for perceived barriers	Resistant to engaging in collaborative activities	0.84
	Questioning the extent of collaborative activities	0.85
	Reticent about revealing personal information	0.85
Scores for cues to action	Information from media	0.72
	Government information	0.78
	Personal communications	0.81
Scores for neighborhood association activities	Difficulty performing as leadres at time of disaster	0.75
	Availability of evacuation area	0.54
	Management of evacuation area	0.61
	Visitation by volunteers	0.57
	Ratio of residents residing in the neighborhood for 20 years	0.10
	Voluntary disaster-prevention organization within neighborhood	0.24
	Neighborhood associations' activities to respond to disaster	0.71
Scores for social capital	Trusting others	0.79
	Taking advantage of others	0.65
	Being helpful to others	0.72
Scores for community's sense of coherence	Large event, overcoming it	0.58
	Calm decision making in response to a large event	0.68
	Something acquired through a large event	0.71
	Community problems, solution by the community	0.69
	Community problems, residents' thoughts on solution	0.83
	Communal problems, strengthening trusting relationships	0.72
Scores for individuals' sense of coherence	Finding solutions for problems	0.86
	Values working on problems	0.85
	Understanding and predicting problems	0.87
	Past experience of earthquakes	0.28
	Knowledgeable about roles as a leader during the earthquake disaster	0.78
Scores for knowledge and experiences	Participated in lectures and workshops	0.76
	Participation in disaster prevention training	0.56
	Knew areas and process for evacuation	0.58
	Knowledgeable about what to be prepared for in normal times.	0.70
	Proactive about non-professional volunteer activities	0.08
	Had concerns about non-professional volunteer activities	0.05
	Experience of volunteering	0.38

Table 3 shows the results of multiple regression analysis of collaborative activity scores and each factor score. Collaborative activities with volunteers was significantly and positively associated with perceived severity (standardized partial regression coefficient β = 0.224, p<0.001) and activity within a neighborhood association (β = 0.539, p<0.001). Also, marginally significant positive relationships were found with SOC in a community (β = 0.137, p = 0.06), SC (β = 0.119, p = 0.08), and perceived benefits (β = 0.116, p = 0.09).

**Table 3:** Results of multiple regression analysis with collaborative activity as the objective variable.

Explanatory variables	standardized partial regression coefficient1)	p values
Perceived susceptibility	0.108	0.11
Perceived severity	0.224	<0.001
Perceived benefits	0.116	0.09
Perceived barriers	0.041	0.55
Cues to action	0.112	0.11
Activities of neighborhood association	0.539	<0.001
Social capital	0.119	0.08
Community's sense of coherence	0.119	0.06
Individuals' sense of coherence	0.119	0.77
Knowledge and experience	0.119	0.18

1) Adjusted for damage circumstances and number of households.

## Discussion

### Perceived severity

This study found that leaders that had higher perceived severity of an earthquake disaster in normal times conducted more collaborative activities with volunteers. Given that perceived severity items concerned damage estimates, collaborative activities may have been influenced by pre-earthquake damage estimates considered by the leaders. Previously in October 2004, the 6.8 magnitude Niigata Prefecture Chuetsu Earthquake had struck in the region surveyed in this study. In a previous study, as actual cases have been shown to exert greater psychological influence than statistical figures [18], leaders may be linked to estimate post-earthquake damage by past experience, perhaps leading to collaborate more with volunteers after the earthquake.

Leaders may also have collaborated with volunteers after the earthquake because they knew the publicly reported extent of quantitative damage, and predicted that they themselves would suffer damage. In Japan, the Cabinet Office and local municipalities estimate total damage in earthquake scenarios, given the size of the quake, the number of collapsed buildings, the number of deaths, and the number of evacuees. Information about damage estimates are released on the Internet and in fliers and newsletters. Residents can get a general grasp of the risks of earthquakes for widespread residential districts. In recent years, hazard maps showing such risk information have been released to the public.

However, a 2002 poll in Japan revealed that 68% of the people surveyed had never actually seen a hazard map [19]. According to the present study, it is considered that the majority of residents will need to be able to anticipate the damage that might occur and do so in normal times, so that collaborative activities with volunteers can be carried out during earthquake disasters. For this to happen, public institutions will need to use a variety of ways to provide residents with official damage estimates, hazard maps, and information on past earthquake disasters, so that they can learn more than they have in the past. In addition, residents can make specific damage estimates for themselves by generating hazard maps and disaster prevention maps that show risks and disaster prevention information for their residential district. Finally, public institutes and local organizations can conduct disaster drills and education based on anticipating earthquake damage. Through the participation of residents in these activities, they can anticipate damage and may be able to carry out a more effective response to earthquake disasters, which includes collaborative action with volunteers.

### Neighborhood association activities

In the present study, neighborhood associations more active in their response to the earthquake disaster were found to have carried out more collaborative activities with volunteers. Neighborhood association members live in the same community, help each other, work together to resolve issues, and have common interests [20]. Neighborhood associations function widely, as SC [21], and serve as community organizations in the public domain addressing such issues as environment beautification and crime prevention [22]. The neighborhood association leaders are central to coordinating disaster prevention activities on a regular basis, and responding to disasters when they occur. The present study found that 98.6% of neighborhood associations responded after the earthquake. In a preliminary survey, associations confirmed the safety of residents, confirmed housing damage, gained an understanding of resident problems and needs, and distributed aid supplies [23].

In regard to the roles of the associations and circumstances of their members, it may be difficult for associations to manage the large number of issues they should respond to in times of disaster. Although all households are expected to join the association, participation rates have fallen recently, and members are growing older [22]. In fact, the massive damage caused by the Great Hanshin-Awaji Earthquake crippled neighborhood association efforts. Volunteer groups located outside the disaster area therefore stepped in to respond [24]. Neighborhood associations alone could not bring the situation under control and so they may have cooperated with large numbers of volunteers in light of this. In the future, residents must consider a neighborhood association response that includes the acceptance of aid from volunteers, and residents must participate in disaster drills and education that includes accepting aid from volunteers. This might permit collaborative activity with volunteers in the early phase of an earthquake disaster.

### SOC in the community

The present study found that more collaborative activities were conducted with volunteers when leaders have a stronger SOC in the community. SOC is a stress-coping ability, consisting of “comprehensibility”, “manageability”, and “meaningfulness” [25]. Antonovsy suggests that, when an entire group, such as a small local organization, encounters a collective stressor, the strength of SOC in a group is more important than individual SOC for dealing with the problem [25]. The present findings that strength of SOC in a community was linked more with willingness to carry out collaborative activities with volunteers are consistent with this. Also, he suggests that in a group with stronger SOC, many individual members tend to perceive the group as comprehensible, manageable, and meaningful [25]. For collaborative activities with volunteers at the time of a disaster, it is necessary for SOC in the community to be stronger. To achieve this, residents need to consider responses to earthquake disasters in normal times, and all community residents should know the results of these determinations.

### SC

In the present study, there was a tendency toward more collaborative activities with volunteers the higher the SC was. Therefore, SC should be built for residents to collaborate with volunteers. Putnam defines SC as characteristics of societal organizations, namely “trust”, “social norms”, and “networks”, which can improve the effectiveness of society by facilitating collective action [26]. It has been suggested that accumulation of SC during non-emergency times for a strong social structure that can withstand disaster is a determining factor for action [27]. Furthermore, research from the United States has indicated that the relations among members of neighborhood associations are influenced by duration of residence, community safety, and education [28]. High SC may be created by facilitating interactions between residents and by regular participation of the majority of residents in neighborhood association events and festivals that are cooperative activities, disaster drills and education, and organized activities.

### Perceived benefits

The present findings showed that the more the participants perceived the volunteer activities as being beneficial, specifically in terms of effectiveness and usefulness, the more they engaged in collaborative activities. In a previous survey, 72% of victims reported that volunteer activities at the time of the disaster contributed immensely to the response for recovery and reconstruction efforts, and 74% were extremely appreciative of volunteer activities [29]. However, public evaluation of volunteers in normal times included, “They don't stick with it,” “They act irresponsibly since they are not being paid,” and “An individual's actions cannot be questioned because they are not part of an organization” [30]. In addition, victims may perceive volunteers as “outsiders,” even during the disaster [6]. Therefore, it is important that residents have accurate knowledge about volunteers and their activities, and understand that collaborative activities with volunteers are effective after an earthquake. It may require that public institutions, local organizations, and neighborhood associations conduct disaster prevention education for residents about collaborative activities with volunteers and carry out disaster prevention drills that include collaborative activities in which volunteer assistance is actively accepted. On the other hand, Britton suggests that the support activities that an average citizen who has not undergone training could provide could in fact worsen situations [31].Volunteers should carry out valuable work during the disaster, so that many people would have strong benefits of volunteers. Thus, education and training on disaster prevention in normal times may be necessary for to develop the potential and skills of those who are willing to engage in volunteers work during a disaster.

There are several limitations to the present study. First, recall bias and lack of accuracy are possible since the survey was conducted a year after the earthquake disaster, on information before and during the disaster. It is extremely difficult to conduct a two-time survey on the same area before and after an earthquake, or in the early phase of an earthquake disaster, as earthquakes occur unexpectedly. This limitation is common to most disaster research. Second, the disaster area, and actions and thoughts of victims were understood solely from the responses of the neighborhood association leaders, in order to relieve victims of the burden of completing the survey. Although the leaders of the neighborhood associations had been selected from among neighbors, and because we had information on neighbors' opinions from past surveys, the actions and thoughts of all victims were not directly surveyed. Third, because the HBM was selected for this study, the survey items were not from existing scales, but were developed by the authors of the present study. Thus there may be some issues related to the validity and reliability of each item [32].

The strengths of the study include the quantified analysis of factors for collaborative activities between leaders, residents, and volunteers, and that it targeted all leaders of neighborhood associations of one city within an extensively damaged area. Furthermore, the findings will be a valuable resource for the development of future strategies and in earthquake disaster research because of the importance placed on the collaborative activities of people during disasters.

In conclusion, collaborative activities with volunteers were significantly related to perceived severity during normal times and to neighborhood association activities during disaster times. Thus, collaboration of disaster victims with volunteers in response to earthquake disaster may require measures for residents to estimate possible damage and to enhance their response to the earthquake disaster in neighborhood association during normal times. For residents to have such estimation abilities, public institutions should provide information related to anticipated disaster damage and appropriate disaster drills and education. In addition, residents will be expected to collaborate in the creation of a disaster prevention map which shows hazards. Lastly, promoting neighborhood association activities may require the participation of many residents in disaster drills and education in normal time, as well as in discussion of neighborhood activities to be conducted during disaster times that includes the acceptance of aid from volunteers.

## Supporting Information

Figure S1 **A conceptual framework leading to the collaborative activities using Health Belief Model.** Becker et al.'s model was referred for the conceptual framework of the present study. The upper row shows the HBM factors reported by Becker et al. [11], while the lower row shows the HBM factors reported in the present study.
(TIFF)

Table S1 **The reliability (Cronbach's alpha value) for each factor score.**
(XLS)

Table S2 **The criterion-related validity for each factor score.**
(XLS)
